# The effect of training and supervision on primary health care workers’ competence to deliver maternal depression inclusive health education in Ibadan, Nigeria: a quasi-experimental study

**DOI:** 10.1186/s12913-021-07208-3

**Published:** 2021-11-30

**Authors:** Adeyinka Olufolake Adefolarin, Asiki Gershim, Arulogun Oyedunni Sola, Gureje Oye

**Affiliations:** 1 Department of Psychiatry, College of Medicine, University of Ibadan, Ibadan, Nigeria; 2 Department of Health Promotion and Education, Faculty Public Health, University of Ibadan, Ibadan, Nigeria; 3 African Population and Health Research Center, Nairobi, Kenya; 4 Department of Women’s and Children’s Health, Karolinska Institute, Stockholm, Sweden; 5 Centre for Entrepreneurship and Innovation, University of Ibadan, Ibadan, Nigeria; 6 WHO Collaborating Centre, Department of Psychiatry, University of Ibadan, Ibadan, Nigeria

**Keywords:** Training, Supervision, Implementation, Maternal depression, Competence, Primary health care workers, Educational materials, Competence

## Abstract

**Background:**

Health workers lack the competence to address maternal depression in the routine health education in Nigeria. Hence, awareness among maternal-child health clients is low. We assessed the effect of training and supervision on knowledge, skills, and self-efficacy of primary healthcare workers in delivering health talks and the clients’ knowledge on maternal depression.

**Methods:**

A quasi-experimental study design was adopted. Five Local Government Area (LGAs) in the Ibadan metropolis were grouped according to geographical proximity and randomly assigned to experimental (Group A = two LGAs) and control (Group B = three LGAs) with 12 primary health centres in each group. All primary health care workers recruited in group A received a one-day training on maternal depression. Good Knowledge Gain (GKG), Good Skill Gain (GSG) and Self-Efficacy (SEG) were assessed in both groups. 1-week post-training, the knowledge of all the PHCs’ attendees in the two groups was assessed. Two weeks post- training, a half of experimental group’s PHCs received supportive supervision and a clinic-based health education delivery skill assessment was conducted. The knowledge of clients and their health seeking were also assessed. Fisher’s exact test, independent t test and Poisson regression were used to analyze differences in percentages and mean/ factors associated with GKG, GSG and SE, using SPSS 25.

**Results:**

Training improved gains in the experimental versus controls as follows: GKG (84.3% vs. 15.7%), GSG (90.7% vs 9.3%) and SEG (100% vs 0%). Training contributed to the good gain in knowledge (RR = 6.03; 95%CI =2.44–16.46; *p* < 0.01); skill (RR = 1.88; CI = 1.53–2.33; *p* < 0.01).) and self-efficacy (RR = 2.74; CI = 2.07–2.73; *p* < 0.01). Clients in the experimental group had higher knowledge gain score than in the control (7.10 ± 2.4 versus − 0.45 ± 2.37); *p* < 0.01). The rater supervisor observed better motivation in the supervised group than the not supervised. Forty clients sought help in the intervention group while none in the control group. Thirty-five clients sought help in the supervised group while only five did in the not supervised.

**Conclusions:**

Training followed by supervision improved the competence of health workers to transfer knowledge to clients. This intervention is recommended for primary healthcare settings to improve uptake of maternal mental health services.

**Supplementary Information:**

The online version contains supplementary material available at 10.1186/s12913-021-07208-3.

## Background

Maternal depression is a public health problem of global concern [1], disproportionately affecting the low and middle income countries (LMICs) [2]. There is a higher prevalence of maternal depression in LMICs reporting up to 65% in some settings compared to 17% reported in the high-income countries. The burden in the LMICs raises concerns as depression is reported as the second leading cause of disability and is projected to be the leading cause of suicide by 2030 [3]. This fact will possibly enlist depression as the most detrimental illness among all mental disorders and non-communicable diseases. Available literature already shows that effects of depression are worse during pregnancy or after childbirth. It leads to poor quality of life for the woman, poor birth outcomes and poor child developmental outcomes [4].

In the LMICs including Nigeria, maternal depression is referred to as “a hidden burden” [1], because it is underdiagnosed, under-treated and there is lack of population awareness about the condition. There was an initial view that maternal depression did not exist in Africa because of strong family social support systems that are protective [5, 6]. However, this no longer stands as migration is breaking down the family support system and there is no structured social support in place [7]. Also, more evidence of high prevalence is emerging from studies which have used culturally adaptable tools [1]. Therefore, available evidence shows that the prevalence of maternal depression in Africa is high and the consequences are equally bad [8]. However, awareness at the population level is still low [9]. There is a need for primary health care workers to offer health education on mental health to women during their attendance of maternal and child health services in primary health care clinics. However, the pre-service training on mental health issues at undergraduate and graduate level is not adequate [10].

In Nigeria, health education is a routine service provision at the primary health care level, but mental health education is not inclusive. This is attributed to lack of competence among primary health care workers to deliver culturally appropriate maternal depression inclusive health education [11]. However, with the increasing decentralization of mental health service to primary health clinics, there is an established need to build the capacity of non-specialist primary healthcare workers to provide mental health service through training and supervision [12].

In this study we focused maternal mental health education, we utilized training and supervision and hypothesized that this would improve knowledge, skill, and self-efficacy of primary health care workers in maternal depression inclusive education delivery. We also evaluated how their performance influenced clients’ knowledge.

## Methods

### Study design and study setting

A two-group prestest-posttest quasi experimental study was conducted from October to December 2016 in Ibadan, Oyo state. The federal system of government in Nigeria functions at three tiers: national, state, and local governments. The Local Government Area (LGA) is primarily responsible for routine primary health care services. The primary healthcare care centres (PHC) are the first level of contact with the community [13]. Ibadan is in the South Western part of Nigeria where the indegenes are the Yoruba speaking tribe who are predomonantly farmers, petty traders and artisans. The total land area of Ibadan is 3123 km2, 15.0% of which is urban, and the rest classified as peri-urban. It has 11 LGA (pronvices). This study took place in 5 LGAs in the metropolis. The other 6 LGAs from periurban and rural areas were excluded. The population distribition in the 5 LGAs selected for the study are Ibadan North (IBN-306,795), Ibadan North-West (IBNW-152,843). Ibadan South-East (IBSE- 266,046). Ibadan South-West (IBSW-282,585) and Ibadan North-East (IBNE-330,399).

This study took place at the comprehensive health centres known as primary health centres (PHCs) in the ward system. A ward is the smallest political structure, consisting of a geographical area with a population range of 10,000 to 30,000 people served by at least one PHC. There are approximately 10 wards in one LGA. The LGA has a department of health which coordinates the administration of the primary health care. At the time of this study, only one doctor was in charge of coordinating all the primary health care in each LGA. At the PHC level, laboratory testing, pharmacy, minor surgery, 24 h service, maternal-child health service (antenatal, delivery and immunization), general health care, community outreaches, admission and referral services are delivered by nurses and midwives as well as some cadres of community health workers. Below the PHC level are two levels: primary health clinics which offers only maternal child–health care, treatment of malaria and other common diseases to a set of villages with a population of 2000–5000 people, and health posts providing only community outreach, maternal child health service, and the dispensing of medication which serves approximately 500 people [14].

### Selection of intervention LGAs and PHCs

The intervention took place in two of the 5 LGAs in the metropolis (Fig. 2). To select a group of LGAs for the intervention, the LGAs were first grouped according to geographical proximity and number of PHCs. Two adjacent LGAs: IBN and IBNW which had 12 PHCs were combined as group A and three LGAs IBSW, IBSE and IBNE also with 12 PHCs were grouped together as B.

These two groups were then allocated randomly into experimental group (A) and control group (B) through a public lottery. In brief, the two groups were labeled on a piece of paper as group A and B, rolled into large balls placed inside a concealed box. It was agreed that the first ball to be picked would be considered as the experimental group and the one remaining in the box would be the control. After shaking the box, a community leader who knew nothing about the study was called to pick one ball from the box. It is important to note that the LGAs were independent, and a worker in public service in Nigeria, can only be a worker in only one public workplace, Monday to Friday and during the intervention delivery, no transfers of staff were made from one LGA to another.

#### Intervention

The intervention used the apprentice model [15], which combines training and supervision in a sequential order. The concept consists of selection of trainees with specific qualifications, classroom teaching, a real-world implementation of learned subject, supportive supervision and evaluation. The objective of the training was to build the competence of primary health workers to deliver culturally appropriate health talk on maternal depression [11]. We (A.O, O. S, and O) developed a training guide in supplementary Table 1, which has the details of the training program activities (the objective, focus of each topic, method, materials, and evaluation). The method of delivering the training included lecture, role play, brainstorming and return demonstration (supplementary Table 1). A supportive supervision guide developed by WHO was used [16, 17]. The validated health education aid provided for the trainees (primary health care workers) included poster, song, leaflet (available in Yoruba, English and Pigin english languages) and a sample of maternal depression health talk (guide) to include iin routine health education. They were developed by researchers A. O and O. S and the training was facilitated by them.

### Training

The training was a one-day training of PHC workers in a classroom with each session allocated a maximum of 20 min on the training program (supplementary Table 1). The participants were divided into two classrooms to maximize the level of interaction with the trainers. Each classroom was monitored by a researcher (A.O or O.S) and each class received the same training by the same facilitator. The focus of the training was mainly on culturally acceptable terms for maternal depression and addressing myths and misconceptions around maternal depression. These were the gaps found in preliminary studies; need assessment carried out among the Primary heath care workers [9] and the perception of maternal depression among maternl child health service users and their informal caregivers in the community [11]. The objective of the training was to build the competence of PHC health worker to deliver explicit health education on maternal depression. The content of the training was developed based on the feedback from the preliminary study on the perception of maternal depression among mothers and their informal maternity caregivers in the community, the knowledge of the definition, of the symptoms, the risk, the consequences of maternal depression, help seeking and the coping with it in supplementary Table 2. The skill training on health talk delivery focused on delivering culturally acceptable terms for maternal depression in the community, clearing misconceptions around perceived susceptibility, perceived risk factors, perceived consequences of maternal child- health clients about maternal depression and help seeking based on the concept of health belief model [18]. The HWs were also trained on the self-efficacy to use IEC materials (poster, song, and leaflet) during health talks.

### Experimental arm

The training was conducted in the second week of October 2016. After training, all the trained participants were scheduled by their PHC nurse administrators to provide maternal depression inclusive health education in their respective PHCs.

In order to evaluate the intervention effect of training, immediate pre-post training evaluation of primary healthcare workers’ knowledge, skills and self-efficacy assessments were conducted as a primary outcome. In the third week of October through the fourth week, an assessment of clients’ knowledge of maternal depression pre-post maternal depression inclusive health talk was also carried out in the 24 PHCs (both experimental and control PHCs) by 4 Research Assistants Y, Z, X, Q, respectively. Four of them were blinded to the allocation of experimental and control group during the training intervention, but they were involved in the training logistics.

### The control arm

The control group did not receive any training but they provided usual health education to their clients which included education on nutrition in pregnancy, importance of routine investigations, prevention of malaria in pregnancy and birth preparedness.

### Supervision

Two female supervisors R and T with a mean age of 34 ± 3.2 years, graduated with Bachelor of Science in nursing degree were recruited. R was trained with the supportive supervision guide in supplementary Table 3, and she was assigned a supportive supervisor’s role while T was a rater supervisor. Supervisor R supervised the Experimental PHCs assigned for supervision alone while the rater T rated both the supervised and the not supervised. Both were blinded to each other.

#### Study participants, sample size

The primary participants were the PHC health workers in the 5 LGAs who provided group health education These included the chief nursing officer, community health officer and community extension worker distributed in the LGAs as follows: IBN (6 PHCs, 72 staff), IBNE (6 PHCs, 53 staff), IBNW (4 PHCs, 45 staff), IBSW (4 PHC, 35 staff) and IBSE (4 PHCs: 55staff). However, health workers who had less than 10 years to retirement and those who were on leave were excluded. The reason for excluding staff within10 years to retirement was that they rarely take part in health education as most of them assume administrative roles. This information was gathered from the LGAs. The clients attending maternal-child health clinics (both antenatal and immunization clinics) were considered the ultimate beneficiaries.

We used the sample size formula for comparing two proportions [19] to estimate the sample size for health workers who participated in the classroom training intervention.

$$ \mathrm{N}=\frac{{\left(\mathrm{Z}\upalpha +\mathrm{Z}\upbeta \right)}^2\mathrm{x}\ \left\{\ {\mathrm{P}}_1\left(1-{\mathrm{P}}_1\right)+{\mathrm{P}}_2\left(1-{\mathrm{P}}_2\right)\right\}}{{\left({\mathrm{P}}_1-{\mathrm{P}}_2\right)}^2} $$

We assumed that P_1_, 50% proportion in the control group will score above average since no previous local studies which utilized proportion were available. The P_2_ was assumed to be 75%, the proportion of respondents with good knowledge, (score above average). The effect size which is the difference P_1_ and P_2_ was assumed to be 25%. The constant Zα^2^ = 1.96, Zβ^2^ = 0.84. The calculated N is 54.91 ≈ 55 for each group. Adjusting for 10% non-response gives a sample size of 60.5. Each group had 60 participants per study arm. The calculated sample size 60 was distributed into the 2 LGAs in the experimental group while 60 distributed in the 3 LGAs in control proportionate to the total number of health care workers in each PHC.

A convenient sample size of 50% of clients were selected for knowledge assessment before and after training of primary healthcare workers and was repeated in the supervised and un-supervised PHCs also.

#### Procedure of data collection

Four female Research Assistants (RAs); Y, Z, Q and X with higher diploma degree and research field work experience were recruited. They were trained for 5 days on how to administer a questionnaire to women attending maternal child health clinics and to run errands for training logistics (Supplementary Table 2). The RAs were blinded from the study protocols and the study arms. The supervisors were trained separately from each other and from the RAs for 3 days (3 h per day) and they were assigned the role of supervision (R supportive supervision and T rater supervisor).

### Training of RAs and supervisors

The RAs Y, Z, Q, and X were trained primarily to administer questionnaire to maternal-child health clients, to assess their knowledge of maternal depression pre-post exposure to health education. The training lasted for 5 days because it included the pretest of the maternal-child clients’ questionnaire. They were trained to pretest the English and the Yoruba version (translated by two bilingual interpreters) of the knowledge questionnaire for clients in PHCs. The back-and-forth review of the questionnaires by the researchers made it to achieve face validity and the pretest process built the capacity of the RAs in the administration of the questionnaire.

Supervisor R and T were blinded to each other’s training and each other’s role. Supervisor (R) was trained using the WHO guide on supportive supervision. Day 1 activities focused on the guide, the important of “no threat or intimidation”. Removal of supervisor’s boundary and encouraging teamwork. Day 2, the content of the objective of the training was communicated and how to achieve it. The use of educational materials and health talk guide, the uniformity of the health talk across all the PHCs was a must. On Day 3, role play of supportive supervision and feedback giving to the PHC health workers were carried out.

For the rater supervisor (T), day 1 activity included familiarization with the study instrument, and question and answer session on the instrument. On Day 2, a role play of the health talk delivery and the rating took place, repeat role plays were carried out until rater (T) understood the approach. The rater was trained to make direct observations on the health talk delivery of the healthcare workers but was not permitted to appear within the view of the health educator and was not expected to make any correction or comment. It was expected to be a silent activity. On Day 3, onsite practical work was carried out at one of the non-metropolises PHC.

### Training intervention data collection procedure

During the training intervention for the PHC workers, codes were assigned to all trainees to track their pre and post knowledge, skill, and self-efficacy’s assessments. Trainees responded to self-administered questionnaire on knowledge and self-efficacy while each of them demonstrated health talk to an arranged group of clients from the secondary health facilities and their skills to deliver maternal depression inclusive health talk was assessed by researchers A. O and O.S.

### Supervision data collection procedure

**Fig. 1:**
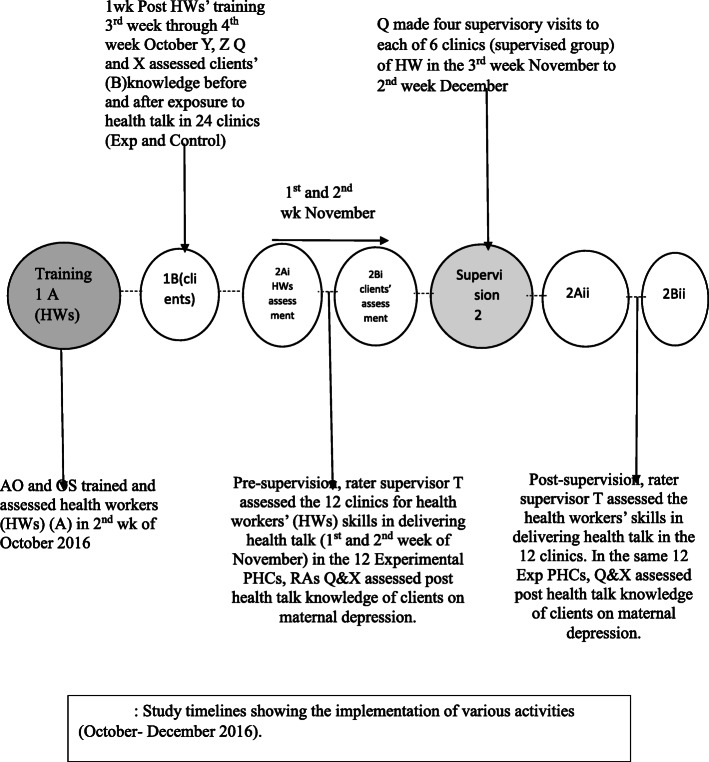
Study timelines showing the implementation of study activities (October–December 2016)

In the 1st and 2nd week of November 2016 (Fig. 1), pre-supervision assessment commenced by two RAs (Q and X) who assessed the maternal-child health clients in all the 12 PHCs in the experimental group. Rater supervisor (T) also carried out pre-supervision assessment of the skill-based health talk delivery on maternal depression inclusive health education. These two RAs (Q and X) and rater supervisor (T) were blinded to the supervision intervention. Supervision commenced in the 3rd week of November 2016 and ended in the 2nd week of December 2016 by supportive supervisor (R) who was blinded to the training intervention and the pre and post-supervision assessment by rater supervisor (T) and RAs (Q and X). The supervision lasted for one-month with six visits to each PHC, which included antenatal and immunization clinics. The RAs (Q and X) repeated the assessment (post-supervision) on the same clients, whom the nurse administrators in the PHCs had scheduled their repeat visit in the second week of December. The rater supervisor (T) also carried out a post-supervision skill assessment of clinic-based health talk delivery in 12 PHCs (both supervised and not supervised). She was also blinded to both the training and supervision interventions. Figure 1 shows the timeline of interventions and evaluations.

### Instrument for data collection and measurements

All study instruments were deveoped based the objective of the study which was based on the gaps found in the preiminary studies by the researchers [18]. For health workers, study instruments included a self-administered 10 scores- item questionaire which was adapted from multichoice questionaire format on depression knowledge [20], similar in content and addressed knowledge gaps identified during the need assesment in the preliminary study [11]. A 15 scores-observational skill checklist was developed from gaps found during the preliminary study on perception about marenal depression [9] and a self administered 20-point self efficacy likert scale questionaire were adapted from the teacher self-efficacy questionnaire format [21]. The questionnaires also contained a section on sociodemographic characteristics. The same 15 scores- observational skill checklist was used by the rater supervsor before and following supervion intervention. All questionnaires were developed in accordance with gaps identified in the preliminary studies and were aligned with the training objectives and were pretested before use. The questiommaires were face- validated by testing each questionaire among a similar population in the PHC outside the study area. The correction of each questionaire and Yoruba translated version went back and forth until the Yoruba and English version had the same meaning. The test-retest reliability was carried out on all the items in each questionaire for absolute agreement using SPSS. The Interclass correlation coeficients were computed for the instruments, and they were considered reliable: knowledge (0.86), skill (0.89), self-efficacy (0.84).

#### Data analysis

The socio demographic data of the health worker trainees was analysed using cross tabulation, frequencies and fisher exact test to compare the characteristics of experimental and control participants. While the sociodemographic charateristics of the maternal child-health clients were analyzed as count and frequency.

The primary outcomes for the training intervention were the propotion of health workers with a good gains in knowledge, skill and self-efficacy respectively. The pre-training scores were subtracted from the post-training scores to compute the gain and the scores for knowledge which is a structured validated multiple choice questionaire, assumed a normal curve, hence the mean (2.4) was considered as cut off. Any score above the mean score was considered as good knowlegde gain. For skill, the questionaire is a checklist used to check the practice of Health workers‘health talk delivery and it has assigned scores. The gain in the skill scores did not assume a perfect normal curve, the median (4.0 at 50 percentile) was used as the cut off, the scores higher that median (4.0 at 50 percentile) was considered as good skill gained and for self-efficacy which is a likert scale, followed the same pattern. Gain in scores more than (4.0 at 50 percentile) were considered good. Then we determined the proportion of healthcae workers with good gain for each of the outcomes. We first explored associations between each sociodemographic factor and the outcome using Fisher exact tests. Factors with p = < 0.01 were then taken into Poisson regression. Since the proportion of participants with good gain was high, we could not use logistic regression to compute odds ratio as this would exaggerate the associations as shown before [22]. We therefore, chose to use poisson regression. First, we conducted bivariate analysis, compute prevalence rate ratios to ascertain the effect of training on the outcome variables, and explored the effect of each of the factors that had a *p* value< 0.01 from the fisher exact test, taking them one by one. Then factors with a likelihood ratio test *p* value < 0.05 were included in a multivariate model [23]. Age was considered an apriori confounder and was included in the final model.

Our secondary outcomes were the clients‘knowledge pre and post training and supervision respectively. The difference of the mean score for these scores was obtained by independent t test since it was a group mean among different clients assessed before and after health education; after training and after supervision. Also, across the supervised and not-supervised PHCs. We used SPSS 25 for the analysis.

#### Ethical consideration

Ethical approval was obtained from the Oyo state ethical review board, ref. no AD 13/479/2016. Letter of permission was also obtained from the PHC coordinators of the 5 selected LGAs. Written informed consents were obtained from health workers, identity was protected, and confidentiality ensured. Study consents were also taken from clients, as they were given the opportunity to withdraw their consent and decline participation even after enrolment.

## Results

### Participants’ recruitment profile

**Fig. 2:**
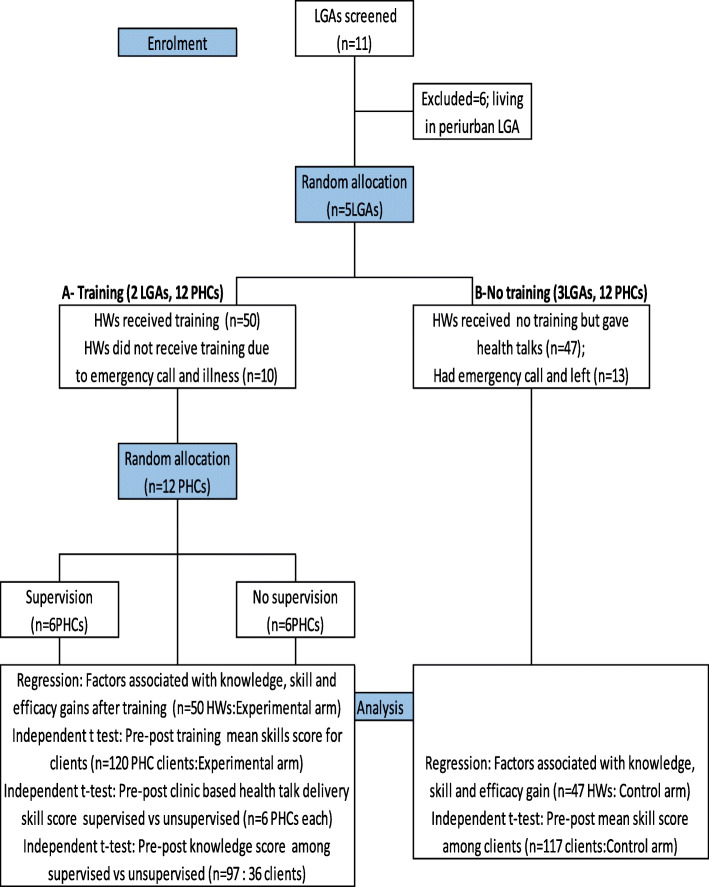
Participant recruitment, randomization and follow up

Figure 2 shows five LGAs were selected for the study after excluding 6 for their location in peri-urban or rural areas. Out of the 60 participants planned to be recruited from 12 primary health clinics per study arm 50 and 47 health workers participated respectively for experimental and control arms. The main reason for non-response was engagement in national immunization campaigns that took place around the period of recruitment (There were no missing values, really, participants did not show up at all). To assess the effect of training of health workers on clients’ knowledge, 120 clients in the experimental arm and 117 from control arm completed the pre and post-test. For the assessment of supervision intervention 97 clients were assessed in supervised arm (Fig. 2).

### Socio-demographic characteristics of health workers and clients who were assessed during the study

As shown in Table 1 the mean age of the health workers in the two arms was similar (mean age 45.4 ± 5.9 years in experimental arm, versus 46 ± 6.1 years for the control arm) and all were Yorubas. Most of the health workers had education up to higher national diploma (HND) level in both arms. The characteristics that differed between experimental and control arm participants respectively were: less proportion of senior nursing staff (24.0% versus 72.3%), more staff with more than 10 years of experience (88% versus 0%), fewer participants with previous training in maternal depression (34.0% versus 59.6%) and more participants involved in health talk delivery control (42% versus 21.3%).

**Table 1:** Comparison of socio demographic characteristics of primary health workers across the experimental group and control group

Sociodemographic characteristics	Experimental arm ***N*** = 50	Control arm ***N*** = 47	Fisher exact ***p*** value
**Age (years)**			
< 45	26 (52%)	27(57.4%)	0.37
> 45	24(48%)	20(42.6%)	
	50(100%)	47(100%)	
**Education**			
< HND^a^	22(44.0%)	13(27.7%)	0.14
> HND	28(56.0%)	34(72.3%)	
	50(100%)	47(100%)	
**Designation**			
CNO^b^/PNO^c^/ CHO^d^	24(48.0%)	34(72.3%)	0.02
CHEW^e^	26(52.0%)	13(27.7%)	
	50(100%)	47(100%)	
**Years of experience**			
< 10 years	06(2.0%)	47(100%)	< 0.01
≥ 10 years	44(88.0%)	0	
	50(100%)	47(100%)	
**Previously trained in maternal depression screening and counseling**			
Yes	12(24.0%)	28(59.6%)	< 0.01
No	38(76.0%)	19(40.4%)	
	50(100%)	47(100%)	
**Involves in Health talk**			
Yes	21(42.0%)	10(21.3%)	0.03
No	29(58.0%)	37(56.1%)	
	50(100%)	47(100%)	

Level of significance *p* < 0.01. *HND*^a^ Higher National Diploma, *CNO*^b^ Chief Nursing Officers, *PNO*^c^ Principal Nursing Officer, CHO^d^ Community Health Officers, *CHEW*^e^ Community Health Extension worker

Supplementary Table 4 shows the characteristics of the clients in both experimental and control arm. The characteristics of the clients in both arms were similar. In brief, the mean age of clients in the experimental and control groups were 29.0 ± 5.6 and 29.3 ± 5.7 years respectively, all were of reproductive age with more than three quarters in the age bracket of 19–30 years, half were pregnant, and the other half were nursing mothers, more than 80% had schooling beyond primary and junior school and less than 10% were unemployed (supplementary Table 4). Supplementary Table 5 Similarly, the clients assessed for effect of supervision form half of the PHCs in the experimental arm have similar characteristics when we compared those in the PHCs where supervision was offered and those not offered.

### Comparison of good gain in knowledge, skills, and self-efficacy between health workers in the experimental and control arms

**Fig. 3:**
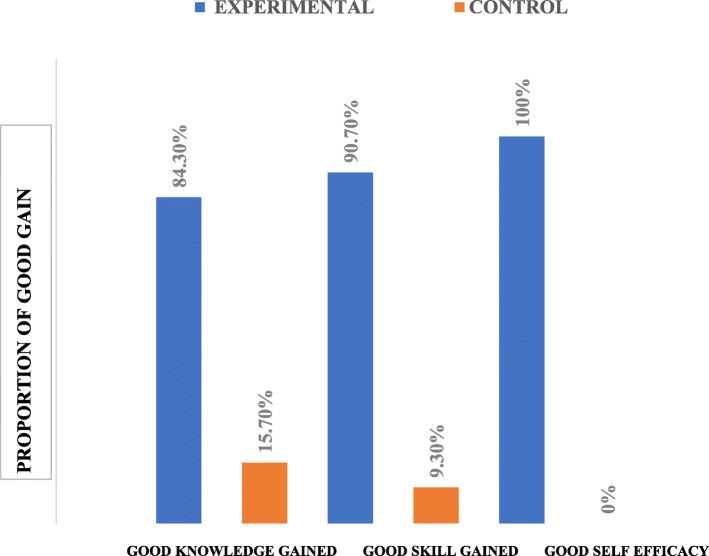
Comparison of knowledge, skills, and self-efficacy gains in experimental and control groups

Figure 3 shows a comparison of proportion of health workers with good knowledge (GKG) good skill gain (GSK) and self-efficacy gain (SEG) assessed in the experimental and control groups;: GKG (84.3% vs. 15.7%), GSG (90.7% vs 9.3%) and SEG (100% vs 0%) respectively. There was a higher proportion of Primary health workers (PHWs) in the experimental group who had good gain in all three outcomes.

### Association of good gain in knowledge, skill, and self-efficacy with characteristics of the health workers

Table 2 shows how the characteristics of the health workers were associated with the good gain in knowledge, skill and self-efficacy only based on the Fisher Exact test. Three factors show statistically significant association with the good gains. These include being in the experimental arm, previous exposure to training on maternal depression and working experience. Designation of staff was associated with knowledge and skill gain but not self-efficacy.

**Table 2:** Factors associated with good gain in knowledge, skill, and self-efficacy of trained primary health care workers

Sociodemographic characteristics	Good gain in Knowledge ***N*** = 97	Fisher exact ***p***-value	Good gain in Skill ***N*** = 97	Fisher exact ***p***-value	Good gain in self-efficacy ***N*** = 97	Fisher exact ***p***-value
Control *N* = 47	8 (15.7%)	< 0.01	4 (9.3%)	< 0.01	0	< 0.01
Experimental *N* = 50	43 (84.3%)		39 (90.7%)		44 (100.0%)	
**Age (years)**						
< 45	26 (50.0%)	0.54	24 (55.8%)	1.00	24(54.5%)	1.00
> 45	25 (50.0%		19 (44.2%)		20(45.5%)	
Total						
**Education**						
< HND	19 (37.3%)	0.84	18 (41.9%)	0.40	19 (43.2%)	0.21
> HND	32 (62.7%)		25 (50.1%)		25 (56.8%)	
**Designation**						
CNO/PNO/ CHO	25 (49.0%)	0.04	20 (46.5%)	< 0.02	22 (50.0%)	0.97
CHEW	26 (51.0%)		23 (53.5%)		22 (50.0%)	
**Experience (years)**						
< 10	14 (27.5%)	< 0.01	9 (20.9%)	< 0.01	5 (11.4%)	< 0.01
> 10	37 (72.5%)		34 (79.1%)		39 (88.6%)	
**Previously trained in maternal depression screening and counseling**						
Yes	13 (25.5%)	< 0.01	32 (74.4%)	< 0.01	10 (22.7%)	< 0.01
No	38 (74.5%)		11 (25.6%)		34 (77.3%)	
**Involve in Health talk**						
Yes	31(60.8%)	0.13	26 (60.5%)	0.19	18 (40.9%)	0.13
No	20 (39.2%)		17 (39.5%)		26 (59.1%)	
	51 (100.0%)					

Level of significance is set at 0.01

### Factors associated with good gain in knowledge, skill, and self-efficacy among the primary health care workers bivariate and multivariate analysis

Table 3 shows results of bivariate and multivariate analysis for association between participants’ characteristics and good gain in knowledge, skill, and self-efficacy. In the bivariate analysis, being in the experimental group and previous exposure to training were associated with good gain in all the three outcomes (all statistically significant). Those who had worked for 10 years, or more were less likely to attain a good gain in knowledge, skills and self-efficacy. Higher cadre staff such as CNO/PNO/ CHO were more likely to attain a good skill gain compared to CHEWs. Age, education level and being involved in a health talk before had no statistically significant associations with all the three outcomes. In multivariate analysis, being in the experimental arm was the only factor that remained statistically significant with the three outcomes with their prevalence rate ratios at 95% Confidence Intervals (RR [95% CI]) as follows: GKG (6.03 [2.44–16.46]), GSK (1.88 [1.53–2.33]) and SEG (2.74 [2.07–2.73]).

**Table 3:** Factors associated with characteristics associated with good gain in knowledge, skill and self-efficacy among primary health care workers at bivariate and multivariate analysis

characteristics	Good knowledge gain (GKG)		Good Skill gain (GSG)		Good Self-Efficacy gain (SEG)	
	Bivariate RR (95CI	Multivariate RR (95%CI)	Bivariate RR(95%CI)	Multivariate RR(95%CI)	Bivariate RR (95%CI)	Multivariate RR (95%CI)
**Study arm**						
Control	1	1	1	1	1	1
Experimental	5.06 (2.51–11.60)	6.03 (2.44–16.46)	2.00 (1.74–2.31)	1.88 (1.53–2.33)	2.41 (2.20–2.65)	2.74 (2.07–2.73)
**Age**						
< 45 years	1	1	1	1	1	1
≥ 45 years	0.86 (0.50–1.50)		1.02 (0.84–1.25)		1.00 (0.82–1.22)	
**Education**						
< HND	1	1	1	1	1	1
≥ HND	1.05 (0.59–1.84)		1.12 (0.91–1.37)		1.15 (0.94–1.41)	
**Designation**						
CHEW	1	1	1	1	1	1
CNO/PNO/ CHO	1.55 (0.89–2.69)	1.76 (0.74–4.57)	1.28 (1.05–1.56)	1.06 (0.88–1.27)	1.20 (0.98–1.47)	0.95 (0.84–1.07)
**Experience**	1	1	1	1	1	1
< 10 years						
≥ 10 years	0.47 (0.27–0.82)	1.81 (0.70–5.18)	0.58 (0.48–0.69)	0.95 (0.74–1.23)	0.53 (0.44–0.63)	0.97 (0.82–1.15)
**Previously trained in Maternal depression screening**						
No	1	1	1	1	1	1
Yes	2.05 (1.12–4.00)	1.30 (0.69–2.2.62)	1.33 (1.10–1.62)	1.05 (0.90–1.22)	1.41 (1.17–1.71)	1.03 (0.93–1.14)
**Involved in Health talk**						
Yes	1		1		1	
No	1.37 (0.77–2.39)		1.17 (0.94–1.44)		1.21 (0.98–1.49)	

Level of significance set at *p* < 0.05

### Effect of training on the knowledge of maternal depression among clients following the training of primary health care workers

We evaluated the effect of health workers’ training on the knowledge of clients accessing maternal-child health PHC services after 1 week of training health workers. One hundred and twenty clients in experimental PHCs and 117clients in the control PHCs completed pre and post-test. The clients in the experimental group had a mean knowledge score gain of 7.10 ± 2.4 while in the control arm, mean knowledge score gain was −0.45 ± 2.37; t = 24.6; giving a mean difference = 7.55. (*p* < 0.05). In the experimental group 40 clients sought help for depressive symptoms while none sought help in the control group.

### Effect of training followed by supervision on skills of health workers on knowledge of clients on maternal depression

We first compared the skill level of PHC workers to deliver health education in the two arms (Supervised ad not supervised PHCs). Ninety-seven clients in supervised PHCs and 36 clients in not supervised PHCs completed pre and posttest. We found a statistically significant difference in the mean scores of PHCs supervised and those not supervised (5.67 ± 5.4; 3.33 ± 2.4) respectively giving a mean difference 2.33; (*p* < 0.05). Rater supervisor observed higher motivation in the supervised PHCs. The clients in the supervised PHCs had mean gained knowledge score 2.75 ± 2.9 and those from PHCs not supervised had a score mean gained knowledge score of 1.67 ± 2.4; t = 1.984 giving a mean difference of 1.09. *p* = 0.06. Twenty-eight clients sought for help to depressive symptoms in the supervised group while only 5 sought help for depressive symptoms in the not supervised group.

## Discussion

In this study, we used one-day training followed by one-month supportive supervision to improve the quality of health talks delivered on maternal depression by primary health workers in the primary health care centres in Ibadan Nigeria. We found that the primary healthcare workers were able to gain good knowledge, skill, and self-efficacy after training. Supervision led to significant improvement in clinic-based skill to deliver explicit health talk on maternal depression. The knowledge on maternal depression was also transferred successfully to pregnant women and nursing mothers who accessed the health talks in the PHCs. Although, previous training in maternal depression showed an association with gain in knowledge, skills and self-efficacy in the bivariate analysis, this association was attenuated at multivariate analysis. This goes to explain that the training offered in this study was likely superior to previous trainings which must have been less specific in content for maternal depression health talk delivery. Possibly, this could also be because mental health training was not adequately taught in health workers’ educational institutions [10, 24]. It is evident in the multivariate analysis that knowledge gain and self-efficacy gain were enhanced by the training offered in the intervention, but skill gain did not change. This goes to emphasize that our intervention was richer in content for knowledge and self-efficacy in delivering health talks on maternal depression than the previous training health workers were exposed to. This means that regardless of previous training, all primary health care workers would benefit from additional training that is structured as our intervention.

The overall purpose of training and supervision is to ensure competence in delivering culturally appropriate services [12]. The evaluation of the immediate outcomes on trained health workers is essentially considered as an evidence that health workers would be able to perform well [25]. Indeed, in our study this was the case, the clients gained knowledge on maternal depression, and they sought help to their perceived depressive symptoms. The good gain among health workers across the three domains of knowledge, skill and self-efficacy outcomes, already indicated good competence to deliver to clients. Mental health issues have diverse health beliefs attached to it across cultures [9, 26], interventions such as ours will go a long way in addressing the knowledge gaps among patients. Training of health workers and empowering them to acquire technical competence to communicate to clients on maternal depression in a way that clients easily understood helped to increase awareness about the condition thus improving uptake of mental health services as shown in our study. In the mental health field, competence to practice is a requirement for non-specialists [12]. These findings are consistent with other studies; in South Africa, 7–8 training sessions improved the knowledge, confidence and attitude of community health workers in counseling of which knowledge was sustained over 3 months [27]. In United Kingdom, a study utilized 3 days training and 4 weeks’ supervision on communication skill in clinical settings, the competence of nurses improved in all the key areas; simulated interview, disclosure of cues and response to cues [25]. A study in Mississippi implemented a complex intervention of community health houses in addressing the control of cardio vascular diseases using a one-month certified training and 1 year supervision among Community Health Workers (CHWs) and this led to a reduction of hospitalization, emergency visits and health care costs reduced [28].

Supervision that followed training in our study had significant effect on the skill of health workers to deliver health education on maternal depression. This is in agreement with earlier recommendations to follow-up a one-day training with supervision [29]. In our study, the supervisor’s observation showed that motivation to provide maternal depression inclusive health education was better in the supervised clinics more than those not supervised. In agreement with these findings is a study by Heaven et al., which deployed 4 weeks of supervision following a three-day training program. Only the nurses who were supervised improved questioning, negotiation, and psychological exploration skills. Contrary to our findings, a study conducted on the quality of care for pregnant women and sick children in 7 LMICs showed no effect of supervision but when training and supervision were combined, there was an improved quality of care in Kenya, Namibia, Malawi, Senegal, Tanzania, Uganda and Rwanda [30]. In Guatemala, Mexico and South Africa, the scale up of the work of trained CHWs in non-invasive cardiovascular testing was very challenging because there was no structured supervision and the political will [31]. Lack of motivation to work and low satisfaction levels among health workers were attributed to lack of structured supervision in Tanzania [32]; A study in Afghanistan showed that regular training without supervision improved the clinical skill of the PHC workers in the integrated management of childhood illnesses but performance was not sustained over time [33], as observed in in India where loss in competence was reported within 3 months of training because of the training was not followed by supervision [34]. In Guinea-Bissau, CHW’s compliance with treatment guideline failed because of lack of supervision [35]. Ultimately, training and supervision should be implemented together to improve and sustain the quality of services received by health care clients [17, 33, 36, 37].

Maternal child health clients were the end beneficiaries of the training and supervision interventions. Our study achieved transfer of knowledge of maternal depression from health workers to clients as demonstrated earlier applying the apprentice model [15]. This improvement in knowledge was further demonstrated by the increase in number of those who sought help to their perceived depressive symptoms. The knowledge of these clients improved on the definition of maternal depression, risk factors and consequences in pregnancy and after birth, coping/ prevention, and help seeking. The outcome was contrary to poor knowledge of maternal depression expressed in form of misconceptions (for example maternal depression is *ogbanje*/*emere* (spirit group membership) in a qualitative study which was earlier carried out among maternal child health clients in the community [9]. We provided a culturally adapted health talk content with the use of educational materials on maternal depression which was locally developed to address the identified misconceptions in preliminary study [1, 9]. Providing health education targeting local beliefs [38] and local practices [39] is key to the success of interventions such as ours in this study. The local adaptability of training and supervision are the emphasis in the global mental health system [12].

Regarding the tools used for assessment of Knowledge, skill and self-efficacy, we used cut–off points based on the distribution of scores in our study population but not cut-off points from other settings (Bloom’s cut-off point). The mean score was used as the cut-off for knowledge because the scores were normally distributed while, the 50 percentile (median score) was used for the skill and the self-efficacy outcomes because they were not normally distributed. This approach is best applied in settings where new tools have been introduced in a study population and where there is no gold standard to compare tools with. Based on the test-retest reliability correlation coefficient values obtained (> 0.70), we could conclude that these tools and our approach to the cut-off points are reliable. We did not come across studies in Nigeria using Bloom’s cut-off point and since we adapted the tools to local context, we made use of the local population distribution to create cut-off points.

Our study has strength in showcasing the effect of training and supervision on the health workers and maternal-child health service users. Locally developed educational materials on maternal depression were used along with the training and the implementation of the health talk delivery. Although, this study was not a randomized control, it employed an experimental study design in the real world setting and provided important findings.

The study had some limitations. First, the intervention and control were not similar in terms of educational qualification attained, years of experience and previous exposure to training on maternal depression. However, this was taken care of by controlling for all these covariates in multivariate analysis. Being a quasi-experimental study, these differences were unavoidable at the design stage. Secondly the clustering effect arising from variability in the LGAs was not taken into account in the sample size calculation, and this could have led to an underestimate of the sample size, however there might have existed little variability among the LGAs since the LGAs selected were all in urban locations and in the same Metropolis. We, therefore, believe the sample size estimate may not have been substantially lower than expected as shown by narrow confidence limits.

Thirdly, the researchers rated the participants’ skills during the training intervention, which could have been rated by a neutral trained rater, hence, there is a possibility that they over-rated the participants’ skills. However, the clients’ knowledge gain observed as a result of the intervention gives us confidence that there was a true effect of the intervention through the healthcare workers. Finally, the self-efficacy ratings by healthcare workers might have been exaggerated, however, we continue to argue that if there was no true gain, then we would not have observed the higher gain in knowledge and help seeking observed among the clients who had benefited from the services of the healthcare workers in the intervention.

## Conclusions

Training improved health workers’ capacity to communicate explicit maternal depression inclusive health education in culturally appropriate way. The follow-up supervision strengthened the skill and the improved the quality of delivering the health education. Hence, maternal-child health clients received quality mental health inclusive health education. Their knowledge improved and they sought help as needed. This implementation can be leveraged to improve maternal mental health service uptake.

## Supplementary Information

**Additional file 1.**

**Additional file 2.**

**Additional file 3.**

**Additional file 4.**

**Additional file 5.**

**Additional file 6.**

**Additional file 7.**

**Additional file 8.**

**Additional file 9.**

**Additional file 10.**

## Data Availability

The data generated and analyzed in this study are available and submitted as additional supporting files.

## Abbreviations

LMIC: Low-and Middle-Income Countries
PHC: Primary Health Care
RA: Research Assistants
CNO/PNO/ CHO: Chief Nursing Officer/Principal Nursing Officer/ Chief Health Officer
CHW: Community Health Worker
LMIC: Low- and Middle-Income Countries
LGA: Local Government Areas
IBN: Ibadan North
IBNE: Ibadan North-East
IBNW: Ibadan North-West, IBNW
IBSE: Ibadan South-East
IBSW: Ibadan South-West
GKG: Good Knowledge Gain
GSG: Good Skill Gain
GSEG: Good Self Efficacy Gain
RR: Relative Risk
