# Supplementary material for: The effect of training and supervision on primary health care workers’ competence to deliver maternal depression inclusive health education in Ibadan, Nigeria: a quasi-experimental study
Source: BMC Health Serv Res. 2021 Nov 30;21:1286. doi: 10.1186/s12913-021-07208-3 (PMC8630868; doi:10.1186/s12913-021-07208-3)
Supplement: Supplementary file 1 — Additional file 1. [file 12913_2021_7208_MOESM1_ESM.docx]

Supplement table 1 Training guide

| **OBJECTIVE:**  **TO TRAIN ON** | **FOCUS OF OBJECTIVE** | **CONTENT** | **METHOD** | | **MATERIALS** | | **EVALUATE** |  |
| --- | --- | --- | --- | --- | --- | --- | --- | --- |
| Feedback from survey and the community on the exploration of maternal depression | To teach on definition of maternal depression + symptoms+ consequences + causes | Every details about maternal depression | | Discussion and return demonstration | | Use talk and picture | Evaluate knowledge, skill and self-efficacy | |
| 1. What Maternal Depression (MD) is | Simple description of maternal depression | Definition | | Talk + use of slides + developed education materials | | Developed education materials+ Literature+ multimedia projector | Pre and Post training knowledge question in the questionnaire and in the skill checklist | |
| 2.Symptoms of maternal depression | Address misconception of local perception. why mental problems is associated with spiritual problem | List of symptoms (scientific and local) | | Talk + use of slides+ pictures+ brainstorming | | Developed education materials+ Literature+ multimedia projector+ info from community+ history of depression | **“** | |
| 3. Consequences of MD | Address misconception of local perception | List of consequences (scientific and local) | | Brainstorming | | Developed education materials+ literature+ multimedia projector+ info from community | **“** | |
| 4.Addressing wrong perception of mothers on the meaning, names, symptoms, consequences and help seeking | Address misconception of local belief gathered from the community | Use facts to address misconception gathered from community about maternal depression | | Talk + use of slides | | Information gathered from community about maternal depression | Pre-post skill check list | |
| 5.Coping/Prevention/  Help seeking and help giving | Address misconception of local perception | List of coping/prevention(gathered from literature and community) | | Talk + use of slides+ pictures | | literature+ multimedia projector+ info from community | **“** | |
