# Supplementary material for: The effect of training and supervision on primary health care workers’ competence to deliver maternal depression inclusive health education in Ibadan, Nigeria: a quasi-experimental study
Source: BMC Health Serv Res. 2021 Nov 30;21:1286. doi: 10.1186/s12913-021-07208-3 (PMC8630868; doi:10.1186/s12913-021-07208-3)
Supplement: Supplementary file 2 — Additional file 2. [file 12913_2021_7208_MOESM2_ESM.docx]

Supplementary table 2: Outline of training

| **Time** | **Activities** | **Resource Person/Coordinator** |
| --- | --- | --- |
| 9.30-10.00 | Registration | Research Assistant Y and Z |
|  | Introduction of participants | Research Assistant Q and X |
| 10-10.20 | Pre-test on knowledge, skill and self-efficacy | O.S and A.O |
| 12.00-12.20 | Why this training? Feedback from perception of mothers and informal care givers about maternal depression and Introduction to maternal depression | A.O |
|  | Tea break |  |
| 12.20- 12.40 | IEC materials on Maternal Depression (MD)( definition, terms, signs, risk, consequences, coping/resilience, help seeking | A.O |
| 1.40-2.00 | Health talk content adaptable to health belief model, checklist and demonstration, | O.S |
|  | Help tips for maternal depression | O.S |
|  | Lunch*(served within lecture)* |  |
| 2.00- 3.00 | Health talk delivery return demonstration using health talk guide | O.S and A.O and al participants |
| 3.00-4.00 | Post-test on knowledge, skill and self-efficacy | O.S and A.O |

Supplementary Table 3

|  | Traditional Supervision | Supportive Supervision (WHO guideline) |
| --- | --- | --- |
| Who performs supervision | External supervisors designated by the service delivery organization | External supervisors designated by the service delivery organization; staff from other facilities; colleagues from the same facility (internal supervision); community health committees; staff themselves through self-assessment |
| When supervision happens | During periodic visits by external supervisors | Continuously: during routine work; team meetings; and visits by external supervisors |
| What happens during supervision encounters | Inspection of facility; review of records and supplies; supervisor makes most of the decisions; reactive problem-solving by supervisor; little feedback or discussion of supervisor observations | Observation of performance and comparison to standards; provision of corrective and supportive feedback on performance; discussion with clients; provision of technical updates or guidelines; onsite training; use of data and client input to identify opportunities for improvement; joint problem solving; follow-up on previously identified problems |
| What happens after supervision encounters | No or irregular follow-up | Actions and decisions recorded; ongoing monitoring of weak areas and improvements; follow-up on prior visits and problems. Encourage chat and phone call reach. |

Source: Comparison of traditional and supportive supervision (Marquez and Kean, 2002), WHO Guidelines for Implementing Supportive Supervision October 2003
