# Supplementary material for: The effect of training and supervision on primary health care workers’ competence to deliver maternal depression inclusive health education in Ibadan, Nigeria: a quasi-experimental study
Source: BMC Health Serv Res. 2021 Nov 30;21:1286. doi: 10.1186/s12913-021-07208-3 (PMC8630868; doi:10.1186/s12913-021-07208-3)
Supplement: Supplementary file 3 — Additional file 3. [file 12913_2021_7208_MOESM3_ESM.docx]

Supplementary Table 3

|  | Traditional Supervision | Supportive Supervision (WHO guideline) |
| --- | --- | --- |
| Who performs supervision | External supervisors designated by the service delivery organization | External supervisors designated by the service delivery organization; staff from other facilities; colleagues from the same facility (internal supervision); community health committees; staff themselves through self-assessment |
| When supervision happens | During periodic visits by external supervisors | Continuously: during routine work; team meetings; and visits by external supervisors |
| What happens during supervision encounters | Inspection of facility; review of records and supplies; supervisor makes most of the decisions; reactive problem-solving by supervisor; little feedback or discussion of supervisor observations | Observation of performance and comparison to standards; provision of corrective and supportive feedback on performance; discussion with clients; provision of technical updates or guidelines; onsite training; use of data and client input to identify opportunities for improvement; joint problem solving; follow-up on previously identified problems |
| What happens after supervision encounters | No or irregular follow-up | Actions and decisions recorded; ongoing monitoring of weak areas and improvements; follow-up on prior visits and problems. Encourage chat and phone call reach. |

Source: Comparison of traditional and supportive supervision (Marquez and Kean, 2002), WHO Guidelines for Implementing Supportive Supervision October 2003
