# Supplementary material for: The effect of training and supervision on primary health care workers’ competence to deliver maternal depression inclusive health education in Ibadan, Nigeria: a quasi-experimental study
Source: BMC Health Serv Res. 2021 Nov 30;21:1286. doi: 10.1186/s12913-021-07208-3 (PMC8630868; doi:10.1186/s12913-021-07208-3)
Supplement: Supplementary file 4 — Additional file 4. [file 12913_2021_7208_MOESM4_ESM.docx]

Supplementary table 4: The sociodemographic characteristics of clinic attendees in the experimental and control groups of training intervention.

| **Sociodemographic characteristics** | **Experimental arm**  **Clients N=120** |  | **Control arm clients**  **N=124** |
| --- | --- | --- | --- |
| **Mean Age (years)** | 29.0±5.6 |  | 29.3±5.7 |
| **Age range** |  |  |  |
| 19-30 | 105 (87.5%) |  | 92(74.2%) |
| 31-40 | 14(11.7%) |  | 32(25.8%) |
| Total | 119 (99.2%) |  | 124(100.0%) |
| **LGA** |  |  |  |
| IBN | 55(45.8%) |  |  |
| IBNE | 65(54.2%) |  |  |
| IBNW | 120(100.0%) |  | 40((32.2%) |
| IBSW |  |  | 40(32.3%) |
| IBSE |  |  | 44(35.5%) |
| Total |  |  | 124(100.0% |
| **Category of women** |  |  |  |
| Pregnant | 60(50.0%) |  | 62(50.0%) |
| Nursing mothers | 60(50.0%) |  | 62(50.0%) |
| Total | 120(100.0%) |  | 124(100.0%) |
| **Education** |  |  |  |
| Pry-JSS | 22(18.3%) |  | 19(15.3%) |
| SSCE | 60(50.0%) |  | 70(56.5%) |
| Post-secondary | 36(30.0%) |  | 34(27.4%) |
| Total | 118(98.3%) |  | 123(99.2%) |
| **Occupation** |  |  |  |
| Unemployed | 7(5.8%) |  | 10(8.1%) |
| Employed | 6(5.0%) |  | 8(6.5%) |
| Self employed | 105(87.5%) |  | 106(85.5%) |
| Total | 118(98.3%) |  | 124(100.0%) |
| **Tribe** |  |  |  |
| Yoruba | 108(90.0%) |  | 110(88.7%) |
| Hausa | 3(2.5%) |  | 9(7.3%) |
| Ibo | 8(6.7%) |  | 3(2.4%) |
| Other tribe | 1(0.8%) |  | 2(1.6%) |
| Total | 120(100.0%) |  | 124(100.0%) |
|  |  |  |  |

120 and 117 completed the pre and post-test in the experimental arm and the control arm respectively
