# Supplementary material for: The effect of training and supervision on primary health care workers’ competence to deliver maternal depression inclusive health education in Ibadan, Nigeria: a quasi-experimental study
Source: BMC Health Serv Res. 2021 Nov 30;21:1286. doi: 10.1186/s12913-021-07208-3 (PMC8630868; doi:10.1186/s12913-021-07208-3)
Supplement: Supplementary file 5 — Additional file 5. [file 12913_2021_7208_MOESM5_ESM.docx]

Supplementary table 5: Sociodemographic characteristics of clinic attendees in supervision and not-supervised clinics

| **Sociodemographic characteristics** | **Clients in the supervised arm**  **N=99** | **Clients in the not-supervised arm**  **N=57** |
| --- | --- | --- |
| **Mean age (years)** | 28.8±5.6 | 27.7±6.2 |
| **Age (years)** |  |  |
| 19-30 | 72 (72.7%) | 25 (43.9%) |
| 31-40 | 26 (26.3%) | 12 (21.1%) |
|  | 98 (99.0%) | 37 (65.0%) |
| **LGA** |  |  |
| IBN | 54(54.5%) | 32 (56.1%) |
| IBNE | 45(45.5%) | 25(43.9% ) |
|  | 99(100.0%) | 57(100.0%) |
| **Category of women** |  |  |
| Pregnant | 19(19.9(%) | 15(26.3%) |
| Nursing mothers | 80(80.8%) | 42(73.7%) |
|  | 99 (100.0%) | 57(100.0%) |
| **Education** |  |  |
| Pry-JSS | 16(56.1%) | 12(21.1%) |
| SSCE | 55(56.1%) | 26(45.6%) |
| Post-secondary | 28(27.5%) | 16(28.1%) |
|  | 99 (100.0%) | 54(94.8%) |
| **Occupation** |  |  |
| Unemployed | 22 (22.2%) | 11(19.3%) |
| Employed | 8 (8.1%) | 4(7.0%) |
| Self employed | 68 (68.7%) | 40(70.2%) |
|  | 98 (99.0%) | 55(96.5%) |
| **Tribe** |  |  |
| Yoruba | 90(2.0%) | 52(91.1%) |
| Hausa | 3(3.0%) | 0 |
| Ibo | 5(5.1%) | 4(7.0%) |
| Other tribe | 1(1.0%) | 1(1.8%) |
|  | 99 (100.0%) | 56(99.9%) |

97 maternal-child health clients in the supervised and 36 in the not-supervised completed the pre and posttests. Attrition was due to urgency to go back to business.
